# *In vitro* Antioxidant Potential of Different Parts of *Oroxylum indicum*: A Comparative Study

**DOI:** 10.4103/0250-474X.65013

**Published:** 2010

**Authors:** S. L. Mishra, P. K. Sinhamahapatra, A. Nayak, R. Das, S. Sannigrahi

**Affiliations:** Institute of Pharmacy and Technology, Salipur, Cuttack, Orissa-754 202, India; 1 Institute of Materials and Minerals Technology, Bhubaneswar, Orissa-751 013, India; 2 St. Peter’s Institute of Pharmaceutical Sciences, Hanamkonada, Warangal-506 001, India

**Keywords:** Free radical scavenging, *Oroxylum indicum*, different parts, polyphenolic compounds

## Abstract

The present study evaluated the *in vitro* antioxidant potential of different parts of *Oroxylum indicum*. 2,2-diphelyl 1-picrylhydrazyl (DPPH), nitric oxide, superoxide anion and hydroxyl radical scavenging potential and reductive ability assay of methanol extract of different parts i.e. root, root bark, stem, stem bark, leaves and fruits were performed. Leaves and bark extracts exhibits highest free radical scavenging activity than bark, stem and fruit extract. Leaves extract showed maximum reductive ability and found to contain maximum amount of polyphenolic compounds. The highest free radical activity may be due to presence of polyphenolic compounds.

Antioxidants are now standing on the mainstay of the treatment and prevention of several diseases.[1-3]. Current research is directed towards finding naturally occurring antioxidants particularly of plant origin. *Oroxylum indicum* Vent. (Bignoniaceae), a rare endangered and threatened medicinal plant widely used traditionally for treating several disorders.[4]. The root-bark is used as an astringent and tonic and also in diarrhoea and dysentery. The stem bark is used in acute rheumatism. In the form of an infusion, it is used as a diaphoretic. The fruits are used as carminative and stomachic, while the seeds are used as purgative. The roots are used in dropsy and the leaves are reputed as an emollient. Tender fruits are described as carminative and stomachic[5]. The root of this plant is also one of the important ingredients in most commonly used ayurvedic formulations like *dantyadyarista, brahma rasayana, dasamula, amartarista, dhanawantara ghrita, narayana taila*[6]. The anti cancer potential of different parts of the plant has already been reported[7,8]. The present study describes a comparative evaluation of different parts of *Oroxylum indicum* for their *in vitro* antioxidant activity.

The different parts of the plant i.e. root, root bark, stem, stem bark, leaves and fruits were collected from the forest region of Orissa and identified at the Institute of Materials and Minerals Technology, Bhubaneswar, Orissa. All the plant materials were shade dried, powdered, sieved and successively extracted with petroleum ether and methanol to obtain the extracts. The each of these extracts was concentrated in a rotary evaporator under reduced pressure, giving individual extracts. Ten milligrams of methanol extract of different parts was dissolved in methanol (1 ml) and solution was serially diluted for antioxidant studies.

Total polyphenolic compounds of different extracts were performed according to the method of Slinkard and Singleton[9]. DPPH radical scavenging activity[10], nitric oxide scavenging assay[11], superoxide scavenging assay[12], hydroxyl radical scavenging assay[13] and reducing power assay[14] were performed as per the standard procedure.

Total polyphenolic content as pyrocatechol equivalent was determined from the standard curve equation of pyrocatechol and presented in Table 1. Root bark and leaves extract were found to contain maximum amount of polyphenolic compounds. The results of different radical scavenging activity are shown on Table 2. Leaves extract also showed maximum reducing power when tested in reducing power assay.

**Fig. 1:**
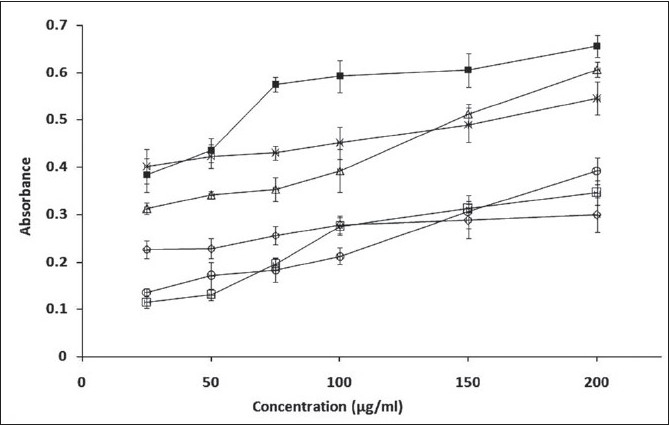
Reducing power of the methanol extract of different parts of *O. indicum* Reducing power is determined by the Fe^3+^-F^2+^ transformation. Values are expressed as mean ± standard deviation of n=3 determinations. Fruit (–□–), stem (–○–), 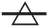 stem bark (–∆–), leaves (–■–), root (–◊–) and root bark (–٭–)

**TABLE 1:** TOTAL POLYPHENOLIC CONTENT OF DIFFERENT PARTS OF *OROXYLUM INDICUM*#

Plant part extract	Phenolics as pyrocatechol equivalents (µg/mg)
RE	35.65±3.25
RBE	105.56±4.78
SE	21.03±2.51
SBE	80.36±5.03
LE	124.7±4.36
FE	21.03±1.15

RE is root extract, RBE is root bark extract, SE is stem extract, SBE is stem bark extract, LE is leaves extract and FE is fruit extract.

# Values are mean± standard deviation of n= 3 determinations

**TABLE 2:** IC_50_ VALUE OF EXTRACT OF DIFFERENT PARTS OF *O. INDICUM* AND STANDARD ANTIOXIDANTS

Scavenging method	IC_50_ value (µg/ml)						
	RE	RBE	SE	SBE	LE	FE	standard
DPPH radical	158.23	112.21	189.36	149.59	106.4	159.46	34.6 (Rutin)
Nitric oxide radical	102.56	89.36	88.15	139.90	72.05	182.25	32.4 (Curcumin)
Superoxide radical	455.36	154.96	681.22	137.30	170.87	399.86	17.84 (Curcumin)
Hydroxy radical	179.35	47.01	309.27	33.30	46.52	76.39	25.85 (Catechin)

RE is root extract, RBE is root bark extract, SE is stem extract, SBE is stem bark extract, LE is leaves extract and FE is fruit extract

Many of the therapeutic potential of the phenolic compounds can be attributed to its antioxidant activity[15]. The result of this study on *in vitro* antioxidant activity of different parts of *O. indicum* reveals that leaves and root bark of the plant have highest radical scavenging activity. Leaves extract was found to contain maximum amount of polyphenolic compounds (124.7±4.36 µg/mg pyrocatechol equivalents) followed by root bark and stem bark extract. Phenolic compounds have been reported to be associated with antioxidative action in biological systems, acting as scavengers of singlet oxygen and free radicals[16]. Leaves extract also showed maximum reductive ability amongst all the extracts. The antioxidant activity of phenolic compounds is mainly due to their redox properties, which can play an important role in absorbing and neutralizing free radicals, quenching singlet and triplet oxygen, or decomposing peroxides[17].

As per the Ayurvedic aspect and reported literature, the root and root bark possess maximum therapeutic potentiality. Since, the plant is an endangered species availability of roots as well as root bark and stem bark is difficult. As per the antioxidant aspect, leaves also can be used instead of root, root bark and stem bark.

## References

[1] Uttara B, Singh AV, Zamboni P, Mahajan RT (2009). Oxidative stress and neurodegenerative diseases: A review of upstream and downstream antioxidant therapeutic options. Curr Neuropharmacol.

[2] Manda G, Nechifor MT, Neagu TM (2009). Reactive oxygen species, cancer and anti-cancer therapies. Curr Chem Biol.

[3] Paravicini TM, Touyz RM (2008). NADPH oxidases, reactive oxygen species, and hypertension: clinical implications and therapeutic possibilities. Diabetes Care.

[4] Warrier PK, Nambiar VPK, Ramankutty C, Warrier PK (2001). Oroxylum indicum. Indian Medicinal Plants.

[5] Chandel KPS, Shukla G, Sharma N (1996). Biodiversity in medicinal and aromatic plants in India: conservation and utilization.

[6] Anonymous (1998). The Ayurvedic Pharmacopoeia of India.Government of India, Ministry of Health and Family Welfare.

[7] Roy MK, Nakahara K, Na TV, Trakoontivakorn G, Takenaka M, Isobe S (2007). Baicaelin, a flavonoid extracted from a methanolic extract of *Oroxylum indicum* inhibits proliferation of a cancer cell line *in vitro* via induction of apoptosis. Die Pharmazie.

[8] Costa-Lotufo LV, Khan MT, Ather A, Wilke DV, Jimenez PC, Pessoa C (2005). Studies of the anticancer potential of plants used in Bangladeshi folk medicine. J Ethnopharmacol.

[9] Slinkard K, Singleton VL (1977). Total phenol analyses: automation and comparison with manual methods. Am J Enol Viticulture.

[10] Blois MS (1968). Antioxidant determination by the use of a stable free radical. Nature.

[11] Marcocci L, Maguire JJ, Droy-Lafaix MT, Packer L (1994). The nitric oxide scavenging property of *Ginkgo biloba* extracts EGb 761. Biochem Biophys Res Comm.

[12] Nishimiki M, Rao NA, Yagi K (1972). The occurrence of superoxide anion in the reaction of reduced phenazine methosulphate and molecular oxygen. Biochem Biophys Res Comm.

[13] Elizabeth K, Rao MNA (1990). Oxygen radical scavenging activity of curcumin. Int J Pharm.

[14] Oyaizu M (1986). Studies on product of browning reaction prepared from glucose amine. Japanese J Nutr.

[15] Srinivasan M, Sudheer AR, Menon VP (2007). Ferulic acid: Therapeutic potential through its antioxidant property. J Clin Biochem Nutr.

[16] Jorgensen LV, Madsen HL, Thomsen MK, Dragsted LO and Skibsted LH (1999). Regulation of phenolic antioxidants from phenoxyl radicals: An ESR and electrochemical study of antioxidant hierarchy. Free Rad Res.

[17] Osawa T, Uritani I, Garcia VV, Mendoza EM (1994). Novel natural antioxidants for utilization in food and biological systems. Post harvest biochemistry of plant food-materials in the tropics.

